# Psychometric validation of the Self-Care Inventory-Revised (SCI-R) in UK adults with type 2 diabetes using data from the AT.LANTUS Follow-on study

**DOI:** 10.1186/1477-7525-11-24

**Published:** 2013-02-26

**Authors:** Leena Khagram, Colin R Martin, Melanie J Davies, Jane Speight

**Affiliations:** 1 AHP Research, Hornchurch, UK; 2 School of Health, Nursing and Midwifery, University of the West of Scotland, Ayr Campus, Beech Grove, Ayr KA8 0SR, USA; 3 Diabetes Research Unit, University of Leicester, Leicester Diabetes Centre, Leicester General Hospital, Leicester LE5 4PW, UK; 4 The Australian Centre for Behavioural Research in Diabetes, Diabetes Australia - Vic, Melbourne, Victoria 3000, Australia; 5 Centre for Mental Health and Wellbeing Research, School of Psychology, Deakin University, Burwood Victoria, 3125, Australia

**Keywords:** Type 2 diabetes, Psychometric validation, Questionnaire, Self-care, Self-management, SCI-R, AT.LANTUS trial

## Abstract

**Background:**

Achieving optimal outcomes in type 2 diabetes (T2DM) involves several demanding self-care behaviours, e.g. managing diet, activity, medications, monitoring glucose levels, footcare. The Self-Care Inventory-Revised (SCI-R) is valid for use in people with T2DM in the US. Our aim was to determine its suitability for use in the UK.

**Methods:**

353 people with T2DM participated in the AT.LANTUS Follow-on study, completing measures of diabetes self-care (SCI-R), generic and diabetes-specific well-being (W-BQ28), and diabetes treatment satisfaction (DTSQ). Statistical analyses were conducted to explore structure, reliability, and validity of the SCI-R.

**Results:**

Principal components analysis indicated a 13-item scale (items loading >0.39) with satisfactory internal consistency reliability (α = 0.77), although neither this model nor any alternatives were confirmed in the confirmatory factor analysis. Acceptability was high (>95% completion for all but one item); ceiling effects were demonstrated for six items. As expected, convergent validity (correlations between self-care behaviours) was found for few items. Divergent validity was supported by expected low correlations between SCI-R total and well-being (r_s_ = 0.02-0.21) and treatment satisfaction (r_s_ = 0.29). Known-groups validity was partially supported with significant differences in SCI-R total by HbA_1c_ (≤7.5% (58 mmol/mol): 72 ± 11, >7.5% (58 mmol/mol): 68 ± 14, p < 0.05) and diabetes duration (≤16 years: 67 ± 13, >16 years: 71 ± 12, p < 0.001) but not by presence/absence of complications or by insulin treatment algorithm.

**Conclusions:**

The SCI-R is a brief, valid and reliable measure of self-care in people with T2DM in the UK. However, ceiling effects raise concerns about its potential for responsiveness in clinical trials. Individual items may be more useful clinically than the total score.

## Background

Optimal management of type 2 diabetes (T2DM) involves a combination of self-care behaviours, e.g. regulating carbohydrate, calorie, fat and alcohol intake; being physically active; taking oral medications as recommended; monitoring blood/urine glucose levels; checking feet. These can be difficult lifestyle changes to make and sustain. The progressive loss of beta-cell function means that people with T2DM are likely to need insulin therapy at some point to achieve and maintain optimal glycaemic outcomes [1]. Despite the biomedical and psychological advantages of adding insulin to the management regimen [2], more than a quarter of people with T2DM would resist the addition of insulin if prescribed [3] and 75% consider initiating insulin a major crisis [4]. This is known as ‘psychological insulin resistance’, which can occur due to fears of hypoglycaemia, weight gain or injections [5]. Many of these concerns and the overall burden of self-care may be minimised with a simpler regimen of a single daily injection, e.g. insulin glargine, which has a longer duration of action, produces more predictable action profile [6], and reduces the risk of hypoglycaemia [7]. Thus, the addition of insulin glargine may add minimal burden to the already complex treatment regimen.

As the vast majority of diabetes care is self-care, performed by the person with diabetes and/or their family/carers, clinicians and researchers need valid and reliable measures of self-care in order to:

– gain insight into the individual’s actual self-care practices

– understand the individual’s barriers to achieving optimal glycaemic outcomes

– understand the burden of self-care experienced and how the individual copes with that burden psychologically

– ensure that treatment is not intensified at a time when the person with diabetes may be already struggling to engage in effective self-care

– to evaluate the outcomes of new approaches to care, e.g. the addition of insulin to the self-care regimen

Yet, there are a number of complexities to the valid and reliable assessment of self-care behaviours and several approaches exist. Clinicians sometimes use glycated haemoglobin (HbA1c) as a proxy measure of self-care, though it is an unreliable indicator of self-care [8]. Objective methods, such as observation (e.g. tablet counts and pedometers), can be costly to implement in studies and clinical practice, and are limited by the individual’s propensity to improve behaviours when monitored [9]. Self-report is the most practical method of ascertaining insights into self-care behaviours but can be subject to bias. The use of specific, nonjudgmental questions, asked in a standardised format reduces the tendency to respond in a socially desirable way [10]. Two commonly used measures are the Summary of Diabetes Self-Care Activities (SDSCA) [11], the Self-Care Inventory [12], and the Self-Care Inventory-Revised [13]. The SDSCA invites the respondent to self-report the frequency of specific behaviours (e.g. “on how many of the last seven days did you take your recommended diabetes medication?”) [11]. The critical issue is how to determine the extent to which the self-report is an accurate reflection of behaviour and engagement with the treatment regimen. The latter is particularly pertinent as the standardised assessment of self-care (i.e. using a questionnaire measure) does not fit easily with a condition such as diabetes, in which each individual is likely to have a different treatment regimen prescribed or is encouraged to take an active role in regulating his/her treatment, adopting a flexible approach (e.g. self-titration rather than fixed insulin doses and mealtimes). Unlike measures that assess the frequency of certain behaviours, the Self-Care Inventory Revised (SCI-R) [9,10] does not presume an “ideal” regimen or that all individuals have the same regimen. Rather, the SCI-R evaluates individuals’ perceptions of how well they engage with their individualised treatment recommendations.

The AT.LANTUS Follow-on study was conducted to follow-up on those who successfully completed the AT.LANTUS Type 2 trial [7] to determine how treatment of their diabetes has evolved since the study finished (e.g. whether or not they remain on glargine, and/or are self-titrating or following a prescribed regimen) and how this may have affected key biomedical and psychological outcomes (e.g. HbA1c, severe hypoglycaemia, weight control, self-care, treatment satisfaction and well-being). Consequently, we needed a self-care measure that would be suitable in the context of an individual’s unique treatment regimen.

The original Self-Care Inventory (SCI) [12,14] has been used in several US studies of T2DM [15,16]. Necessarily, measures of self-care need to be updated regularly to maintain relevance to modern treatments and technologies. A more recent study by Weinger and colleagues revealed that the revised version (SCI-R; modified to reflect current diabetes practice and to be more suitable for adults rather than adolescents) is a psychometrically sound measure of engagement with recommended diabetes self-care behaviours of adults with type 1 diabetes or T2DM in the US [13]. It has been shown to have satisfactory internal consistency reliability (α = 0.87), good evidence of concurrent validity with the SDSCA measure of self-care (r = 0.63) and construct validity, with low to moderate correlations with measures of diabetes-related distress, depression, anxiety and self-efficacy [13]. To our knowledge, no such study has been undertaken in the UK. Thus, the aim of the present study was to undertake further psychometric validation of the SCI-R for use in adults with T2DM in the UK using data from the AT.LANTUS Follow-on study.

## Methods

### Participants

The AT.LANTUS Type 2 study was based in the UK, including approximately 600 participants from 137 centres in the UK. Approval for the study was granted by the Leicestershire, Northamptonshre and Rutland Research Ethics Committee. Methods for the AT.LANTUS trial are reported elsewhere [7]. For sites that agreed to participate in the AT.LANTUS Follow-on study, participants who completed the original trial were approached at clinic visits or sent postal invitations and asked to complete and return a form if they did not want to participate. After two weeks, patients were contacted by telephone to check if they had received the invitation and a visit was booked if they wanted to participate. All participants completed the questionnaires at the clinic during the study visit. A total of 353 participants completed questionnaires and provided biomedical data.

### Biomedical outcomes

HbA1c is a measure of average blood glucose over the past two-three months. An HbA1c was conducted at the visit unless it had been recorded in the medical notes within the past three months, in which case this figure was used. HbA1c was analysed locally using DCCT aligned methods.

### Psychological outcomes

*The Self*-*Care Inventory*-*Revised* (*SCI*-*R*) [12,13] is a 15-item self-report questionnaire assessing patients’ perceptions of various self-care behaviours, i.e. diet (4 items), glucose monitoring (2 items), medication administration (3 items), exercise (1 item), low glucose levels (2 items), preventative/routine aspects of care (3 items). For people with T2DM it is recommended that three items (checking ketones, adjusting insulin and wearing a Medic Alert) are not scored [22]. Respondents rate their own self-care on a 5-point Likert scale to reflect how well they followed recommendations during the past month (i.e. from “never” (scored as 1) to “always” (scored as 5)). For scoring, items are averaged and converted to a 0–100 point scale, with higher scores indicating higher levels of self-care.

*The Well*-*being Questionnaire* – *28 items* (*W*-*BQ28*) [17] is an extended version of the widely used 12-item generic version of the W-BQ [18]. It includes seven 4-item subscales: Generic Negative Well-being, Generic Positive Well-being, Energy, Generic Stress, Diabetes-specific Negative Well-being, Diabetes-specific Negative Well-being and Diabetes-specific stress. Higher scores indicate higher levels of the named aspect of well-being.

*The Diabetes Treatment Satisfaction Questionnaire* (*DTSQ*) [19] includes eight items, six of which form a scale (scored 0–36) in which higher scores indicate greater treatment satisfaction. Two individual items (scored 0–6) measure perceived frequency of hyperglycaemia and hypoglycaemia. Higher scores indicate greater perceived frequency.

### Psychometric analyses

Psychometric validation consists of a series of statistical analyses to determine the acceptability, reliability, validity and responsiveness of a PRO measure. All statistical analyses were performed using SPSS 16.0 or AMOS 16.0. Skewness and kurtosis statistics demonstrated non-normal distributions, indicating use of non-parametric statistical tests.

*Acceptability* was assessed by examining completion rates and identifying floor and ceiling effects (i.e. >25% scoring minimum/maximum response).

*Reliability* coefficients (Cronbach’s α) were calculated for various computations of the SCI-R scale score (see six models discussed below). A Cronbach’s alpha reliability statistic of >0.70 is considered as the minimum acceptable criterion of internal consistency [20].

*Content validity* was assessed by confirmatory factor analyses (CFA) on the structure of the SCI-R. Some of the items are not necessarily suited to T2DM [13] or would not necessarily be expected to contribute to overall self-care (in terms of predicting or correlating with other outcomes, e.g. HbA1c). Thus, we evaluated several combinations of items. Three unidimensional models were tested (excluding various combinations of items) to investigate whether the structure reported elsewhere [13] could be replicated. Item 3 was excluded because it was considered irrelevant to people with T2DM and was completed by only 34% of respondents; item 13 was excluded because wearing a medic alert is unlikely to be associated with other self-care activities or HbA1c; item 15 was excluded because adjusting insulin was unlikely to be relevant to all respondents. The two-factor models were selected on the basis of conceptual relevance, with the intention of identifying a subscale that would have a good fit to the data and be capable of predicting self-care outcomes, e.g. HbA1c. Multiple goodness of fit tests were used to evaluate the fit between the six models and the data [21]. A comparative fit index (CFI) [22] and Tucker-Lewis index (TLI) [23] of ≥0.90 indicate a good fit to the data [24], a root mean squared error of approximation (RMSEA) value <0.08 indicate an acceptable fit to the data [24], while values <0.05 indicate a good fit to the data [25].

*Convergent validity* was assessed by examining Spearman’s rank correlations between the items of the SCI-R. It was expected that there would be moderate correlations (r = 0.3-0.5) between items. Strong correlations were not expected as performing one self-care activity does not necessarily mean another will be performed at a similar level. *Divergent validity* was assessed by examining Spearman’s rank correlations between the SCI-R and the W-BQ28 and DTSQ. Weak correlations (r < 0.3) were expected, as the SCI-R would not be expected to be highly correlated to measures of well-being or treatment satisfaction.

*Known*-*groups validity* was assessed by examining differences (Mann Whitney *U*-test) in SCI-R total scores duration of diabetes (median split), HbA1c (split at 7.5% (58 mmol/mol), the maximum target recommended [23]) and presence/absence of complications.

*Interpretability* can be informed by establishing minimal important differences (MIDs) and minimal clinically important differences (MCIDs) in mean scores, so that the significance of changes in scores following intervention or differences between groups can be understood. The minimal important difference (MID) was calculated using both a distribution-based approach (i.e. defining the MID as 0.5 times the standard deviation) and an anchor-based approach, using DTSQ scores (median split) serving as anchors for the SCI-R scores. An anchor-based MCID was also calculated with HbA1c (split at 7.5% (58 mmol/mol)).

Descriptive statistics are mean ± standard deviation (SD) or (number %).

## Results

### Descriptive data

Of the 353 participants, 137 (38.8%) were women, the mean age was 65.6 ± 9.3 years, the mean duration of diabetes was 16.6 ± 6.6 years and the mean HbA1c was 8.3 ±1.4% (67 ± 16 mmol/mol) (Table 1). Almost one third (104; 29.5%) had at least one long-term complication.

**Table 1:** Demographic and biomedical characteristics (n = 353)

	**Demographic**	**n**	**Mean**	**SD**	**Median**	**Mode**	**Min**	**Max**	**Range**
Age (years)	353	65.6	9.34	66.0	70	18	92	74
Duration of diabetes (years)	352	16.63	6.55	15.91	16.85	5.88	41.73	35.85
Current HbA1c (%)	352	8.31	1.39	8.00	8.00	5.80	13.90	8.10
Current HbA1c (mmol/mol)	352	67	16	64	64	40	128	88
Complications (n)^a^	352	1.36	1.24	1.00	.00	.00	5.00	5.00

SD (standard deviation).

^**a**^retinopathy, neuropathy, nephropathy, macroangiopathy, other vascular disorders.

### Acceptability of the SCI-R

Completion rates were high (>95%) for all items except items 3 and 10. Substantial ceiling effects were apparent for six items (Table 2).

**Table 2:** SCI-R: mean (SD), exploratory factor analysis, acceptability, internal consistency reliability (of uni-dimensional Model 2 (13 items excluding 3 & 13)), and interpretability

**SCI-R items**			**Exploratory factor analysis**		**Acceptability**				**Reliability**		**Interpretability**	
	**Mean (standard deviation)**		**Forced one-factor solution**^**1**^	**Forced one-factor solution**^**1 **^**(excluding item 13)**	**Completion rate N = 353 n (%)**		**% floor**	**% ceiling**	**Alpha**	**Item-total correlations**	**MID**	**MCID**
1. Check blood glucose with monitor	4.26	(0.94)	0.55	0.55	350	(99.2)	1.4	**53.3**	.75	.46	0.26 – 0.47	0.38
2. Record blood glucose	3.86	(1.32)	0.45	0.45	343	(97.2)	8.2	**45.3**	.76	.35	0.34 – 0.66	0.34
3. Check ketones ^a^	2.40	(1.68)	-	-	121	(34.2)	-	**-**	-	-	-	-
4. Correct dosage of pills/insulin	4.81	0.57	0.52	0.54	343	(97.2)	1.1	**82.7**	.76	.36	0.07 – 0.28	0.05
5. Take pills/insulin at correct time	4.60	0.63	0.55	0.56	348	(98.6)	0.8	**64.0**	.76	.38	0.19 – 0.31	0.19
6. Correct food portions	3.77	0.80	0.66	0.67	349	(98.9)	1.7	15.3	.75	.51	0.39 – 0.40	0.08
7. Meals/snacks eaten on time	3.92	0.83	0.69	0.69	348	(98.6)	1.7	22.1	.74	.53	0.29 – 0.42	0.31
8. Keep food records	1.59	0.99	0.39	0.39	346	(98.0)	**62.9**	3.7	.76	.31	0.20 – 0.49	0.05
9. Read food labels	3.12	1.37	0.49	0.49	347	(98.3)	18.7	19.0	.76	.38	0.32 – 0.69	0.14
10. Recommended carbohydrates	3.57	1.24	0.54	0.54	302	(85.6)	8.5	21.0	.75	.44	0.52 – 0.62	0.26
11. Carry quick acting sugar	3.78	1.48	0.54	0.53	347	(98.3)	15.3	**46.7**	.75	.43	0.25 – 0.74	0.26
12. Attend clinic appointments	4.86	0.48	0.57	0.57	347	(98.3)	0.6	**87.8**	.76	.42	0.10 – 0.24	0.13
13. Wear medic alert	2.15	1.71	0.25	-	348	(98.6)	**65.4**	22.1	-	-	0.15 – 0.85	0.14
14. Exercise	3.35	1.09	0.52	0.52	349	(98.9)	5.4	17.0	.75	.40	0.26 – 0.54	0.08
15. Adjust insulin dosage	3.38	1.42	0.42	0.42	338	(95.8)	17.0	**24.9**	.76	.35	0.06 – 0.71	0.04
SCI-R Total Score ^b^	69.00	12.82			348	(98.6)	-	**-**	.77	-	6.50 – 7.0	4.00

^a^As so few respondents completed item 3, this item was excluded from the overall analyses.

^b^SCI-R Total Score = sum of 13 items (excluding items 3 and 13).

*MID*: minimal important difference; *MCID*: minimal clinically important difference.

### Exploratory factor analyses

An unforced principle components analysis (PCA) with varimax rotation produced 5 components with eigenvalues >1. However, neither this nor subsequent forced 4-, 3- and 2-factor models (not shown) produced interpretable factors. For a 13-item forced one-factor solution (items 3 and 13 excluded; Table 2), all items loaded >0.4 on a single factor, except item 8 (keep food records), which loaded 0.39. This item was retained in the scale given its salience for the concept of self-care in T2DM and its factor loading’s proximity to the accepted threshold of 0.4.

### Confirmatory factor analyses

Fit indices for the models tested using CFA are shown in Table 3, with path diagrams shown in Additional file 1: (Appendix 1). The model depicting the SCI-R as a 14-item uni-dimensional scale (excluding item 3) did not provide a good fit to the data. Thus, a number of additional models were tested, each based upon theoretical rationales for how the items of the SCI-R might be expected to relate to each other and form scales. None of the additional models evaluated offered a good fit to the data.

**Table 3:** Model fit of the SCI-R

	**Model**^**1**^	***χ***^***2 ***^***(DF)***	**CFI**	**TLI**	**RMSEA**
1: unidimensional, 14 items (excluding item 3)	412.49** (77)	.663	.602	.111
2: unidimensional, 13 items (excluding items 3 and 13)	385.36** (65)	.670	.670	.118
3: unidimensional, 12 items (excluding item 3, 13 & 15)	349.02** (54)	.677	.605	.125
4: 2-factor model (active-management & preventative routine), 15 items (including item 3)	427.50** (89)	.662	.601	.104
5: 2-factor model (glycemic control & other items), 15 items (including item 3)	429.53** (89)	.660	.599	.104
6: 2-factor model (glycemic control & other items), 13 items (excluding items 3 & 13)	385.36** (64)	.669	.597	.119

** p < 0.001.

^1^As recommended by Weinger et al. (2005) and indicated by substantial missing data, item 3 (check ketones) was excluded from our initial testing of unidimensional models. However, as many people had completed item 3, we tested its inclusion in models 4 and 5.

DF, degrees of freedom, CFI, comparative fit index, TLI, Tucker-Lewis index, RMSEA, root mean squared error of approximation.

### Reliability analyses

For the 13-item scale (excluding items 3 and 13, based upon the factor solution depicted in Table 2), Cronbach’s alpha was satisfactory (α = 0.77). Item-total correlations for the SCI-R ranged from r = 0.31 to r = 0.53 (Table 2).

### Dealing with missing values

Where missing values exist, an SCI-R scale score can be imputed as long as the scale remains reliable (i.e. α > 0.70) with fewer than 13 items contributing to the scale score. The item contributing most to the reliability of the 13-item SCI-R scale (i.e. with the lowest ‘alpha if item deleted’, α = 0.743) was item 7. This demonstrated that even if respondents did not complete the ‘best item’, reliability would remain acceptable. Thus, item 7 was removed and the analysis re-run. The item that contributed most to the reliability of a 12-item SCI-R was item 1 (α = 0.718) demonstrating that the reliability again remained acceptable. Thus, item 1 was removed and the analysis re-run. Item 10 (α = 0.688) contributed most to the reliability of an 11-item SCI-R scale, indicating that reliability would fall below acceptable levels (i.e. α = 0.7) if it were removed (and/or only 10 items were complete). Thus, the SCI-R total score remains reliable if the respondent has completed 11 or more of the 13 items. Further analyses using the SCI-R total score were conducted where respondents completed 11 or more of the 13 items (n = 348).

### Convergent and divergent validity

The convergent validity of the SCI-R was not supported; the highest correlation was observed between ‘recording blood glucose’ (item 2) and ‘checking blood glucose with a monitor’ (item 1), while the majority of the remaining items produced either low correlations or none at all (Table 4). Divergent validity was supported with expected low correlations observed between SCI-R total and W-BQ28 subscales/scales (r_s_ = 0.02-0.22) and DTSQ total (r_s_ = 0.29) (Table 5).

**Table 4:** Convergent validity: correlation between SCI-R items & SCI-R total score

	**SCI-R**	**SCI-R total**	**1**	**2**	**4**	**5**	**6**	**7**	**8**	**9**	**10**	**11**	**12**	**13**	**14**
1. Check blood glucose with monitor	.523^**^													
2. Record blood glucose	.504^**^	.566^**^												
4. Correct dosage of pills/insulin	.320^**^	.150^**^	.190^**^											
5. take pills/insulin at correct time	.284^**^	.157^**^	.114^*^	.473^**^										
6. Correct food portions	.491^**^	.194^**^	.112^*^	.148^**^	.147^**^									
7. Meals/snacks eaten on time	.509^**^	.250^**^	.210^**^	.163^**^	.215^**^	.529^**^								
8. Keep food records	.429^**^	.156^**^	.179^**^	.146^**^	.092	.132^*^	.163^**^							
9. Read food labels	.545^**^	.111^*^	.102	.147^**^	.100	.299^**^	.210^**^	.225^**^						
10. Recommended carbs	.636^**^	.221^**^	.207^**^	.215^**^	.218^**^	.382^**^	.392^**^	.144^*^	.307^**^					
11. Carry quick acting sugar	.552^**^	.179^**^	.129^*^	.166^**^	.056	.133^*^	.198^**^	.135^*^	.256^**^	.304^**^				
12. Attend clinic appointments	.275^**^	.234^**^	.164^**^	.254^**^	.173^**^	.077	.152^**^	.045	.055	.194^**^	.220^**^			
13. Wear medic alert	.171^**^	.112^*^	.083	.067	.050	.023	.114^*^	.052	.134^*^	.045	.248^**^	.087		
14. Exercise	.472^**^	.077	.034	.120^*^	.095	.204^**^	.125^*^	.160^**^	.174^**^	.214^**^	.242^**^	.130^*^	.056	
15. Adjust insulin dosage	.488^**^	.190^**^	.090	-.092	.024	.151^**^	.124^*^	.146^**^	.128^*^	.208^**^	.209^**^	.105	.103	.264^**^

**Table 5:** Divergent validity (Correlation of SCI-R with DTSQ & W-BQ28)

	**SCI-R total**
DTSQ: Total Score	.285^**^
DTSQ: Perceived frequency of hyperglycaemia	-.055
DTSQ: Perceived frequency of hypoglycaemia	.089
W-BQ28: Generic Negative Well-Being	-.139^**^
W-BQ28: Generic Positive Well-Being	.188^**^
W-BQ28: Generic Stress	-.179^**^
W-BQ28: Energy	.020
W-BQ28: Diabetes-Specific Negative Well-Being	-.114
W-BQ28: Diabetes-Specific Positive Well-Being	.206^**^
W-BQ28: Diabetes-Specific Stress	-.173^**^
W-BQ28: W-BQ12 scale	.170^**^
W-BQ28: W-BQ16 scale	.214^**^
W-BQ28: Generic W-BQ12 scale	.213^**^
W-BQ28: Diabetes-Specific W-BQ12 scale	.211^**^

*p < 0.05, ** p < 0.01 (Spearman Rank correlations).

### Known-groups validity

Known-groups validity was supported partially with significant differences in SCI-R total by HbA_1c_ (≤7.5% (58 mmol/mol): 72 [11], >7.5% (58 mmol/mol): 68 [14], p < 0.05) and diabetes duration (≤16 years: 67 [13], >16 years: 71 [12], p < 0.001). No significant differences in the total score or any SCI-R items were found between presence/absence of complications or by treatment algorithm (Additional file 1: Appendix 2).

### Interpretability

Table 2 indicates the MID range and MCID for each item and total score of the SCI-R. The MCID was established as 4.00, indicating that a difference in total score (e.g. between groups or following intervention) of ≥4 points would be clinically significant. Using distribution and anchor-based approaches, a more conservative MID was established, requiring a difference of ≥6.5 points to be considered important. Table 2 shows the broad range of MIDs and MCIDs for individual items, reflecting uncertainty for the significance of differences for individual scores.

## Discussion

This study examining the preliminary psychometric properties of the SCI-R in adults with T2DM in the UK demonstrated evidence supporting its structure, reliability, divergent validity and known groups validity. Although a uni-dimensional scale could not be confirmed using CFA, exploratory analyses supported a 13-item uni-dimensional scale (with satisfactory reliability), consistent with the findings of the US validation [13]. The internal consistency of the 13-item unidimensional scale was satisfactory, and also consistent with the US validation.

Despite identifying a general factor from which a total score can be computed, the lack of convergent validity for the majority of items indicates that they are relatively disparate, confirming previous findings that different aspects of self-care do not correlate highly [26,27], and reflecting the multidimensional nature of diabetes self-care [11].

Indeed, a range of independent behaviours are required for optimal self-management and individuals may choose to undertake certain self-care activities without necessarily taking on others. For example, an individual may record blood glucose results diligently but not think it an important part of his/her self-management to read food labels. This may be due to some aspects of self-care being more/less consistent with others, the value/emphasis placed on each activity by healthcare providers or reflect the variable ease/difficulty of incorporating various self-care behaviours into one’s routine on a regular basis. As has been found with knowledge [28], scores for individual aspects of self-care activities may be more predictive of various outcomes (e.g. HbA1c) than the total score. In light of these findings (and mixed support for a uni-dimensional scale), we recommend that SCI-R items are scored individually as well as summed to form a total score for some purposes.

Despite our findings that uni-dimensionality may not be necessary (or expected) when assessing self-care behaviours in T2DM, the responsiveness (or sensitivity to change) of *individual* items may be an issue. Like other measures of self-care behaviour [11], many items were prone to ceiling effects, which may be a factor of some aspects of self-care being easier or considered more important than others to undertake consistently. This was not reported in the US validation despite similar total scores but the SCI-R was found to be responsive following a psychological or cholesterol-intensive intervention [13]. One of the challenges of using self-report measures of self-care is that they are likely to be prone to social desirability bias (i.e. the individual’s natural tendency to respond to items in a way that he/she believes others would value). Despite being cost-effective and practical, self-report of self-care behaviours is considered by many to be problematic for this very reason. Tools are available to assess the individual’s tendency to respond in socially desirable ways [29,30] but we were unable to use those in the context of our study and they may not be practical in most research or clinical practice scenarios.

Another challenge facing investigators is determining the significance of any observed change in scores, as statistical significance can often be achieved with large sample sizes. Furthermore, when working clinically, it is not possible to ascertain the statistical significance of a difference between scores at two consecutive consultations for a single patient. In response to this challenge, the minimal clinically important difference (MCID) is a statistical technique that can be defined as “the smallest difference in score in the domain of interest which patients perceive as beneficial and which would mandate, in the absence of troublesome side effects and excessive cost, a change in the patient’s management” [31]. The FDA’s draft guidance on the use of patient-reported outcome measures in support of labeling claims [32] encouraged developers and researchers to identify a MID or MCID as a benchmark for interpreting the clinical importance or relevance of study results to patients – though the more recent definitive guidance has omitted this recommendation [33]. In this UK study, the MCID for the SCI-R total scale was established for the first time, indicating that a minimum change of four points would be required for the change to be considered clinically meaningful, though a more conservative MID suggested that a change of >6.5-7 points would be needed.

In support of its divergent (or discriminant) validity, and as expected, we were able to demonstrate that responses to the SCI-R were largely unrelated to measures of treatment satisfaction (DTSQ) and psychological well-being (W-BQ28). Known-groups validity was partially supported. As expected, we found that those with a lower HbA1c (≤7.5% (≤58 mmol/mol)) reported greater engagement in self-care behaviours. We also found that those with a longer duration of diabetes (>16 years) reported greater engagement in self-care behaviours overall. This finding may be due to the fact that those individuals have had more time to adapt positively to living with diabetes. An alternative explanation may be that increased self-care in those with a longer duration is confounded with the development of complications, increasing the individual’s perceived severity of and susceptibility to negative outcomes and, thus, increasing their engagement with self-care activities. However, we found no differences in total self-care between those with and without complications or between the different insulin treatment algorithms.

### Strengths and limitations

The current study offered the opportunity to establish the psychometric properties of the SCI-R in a large cross-sectional study of adults with type 2 diabetes to the UK. While the analyses reported here offer preliminary evidence of validity and reliability, full psychometric validation also involves assessment of test-retest reliability, predictive validity and responsiveness, which requires longitudinal data. This study is also limited by the lack of opportunity for assessing the convergent validity of the measures, i.e. the SCI-R was the only measure of diabetes self-care included in the study. It would have been ideal to assess convergent validity by correlating SCI-R scores with scores on other instruments measuring aspects of diabetes self-care, such as the Summary of Diabetes Self-Care Activities (SDSCA) [8], a measure of the frequency of performing diabetes self-care tasks. Finally, this study was conducted using data from the AT.LANTUS Follow-on study, which followed up on approximately two-thirds of those in the original AT.LANTUS trial. While the demographics and clinical characteristics of those in our sample were similar to those at the end of the original trial, those who agreed to participate in the AT.LANTUS Follow-on study may well have been more likely to follow their recommended treatments, hence contributing to the ceiling effects observed here.

## Conclusions

Notwithstanding the limitations mentioned above, the findings from the present study provide important insights into the suitability of the SCI-R in people with T2DM in the UK, with implications for clinical practice and research. They also extend previous observations [13] regarding the psychometric properties of the SCI-R. Ceiling effects raise concerns about the potential of the SCI-R for responsiveness in clinical trials but this is likely to be true of other measures of self-care, all of which are prone to social desirability bias. Perhaps, more important is the issue of whether or not the SCI-R items should be summed to form a total scale. On the basis of the findings from the present study, and corroborating the US validation [13], we recommend that analysis involves scoring of individual items as well as the total score. Analysis of data from the AT.LANTUS Follow-on trial has demonstrated that the SCI-R is a valid and reliable measure of diabetes self-care and is suitable for use in people with T2DM in the UK.

## Competing interests

This study was funded by sanofi aventis UK. Quanticate managed data collection and data entry for the AT.LANTUS Follow-on study but neither Quanticate nor sanofi aventis were involved in the analyses or interpretation of the data presented here or in the preparation of the manuscript. The authors have no other competing interests to declare.

## Authors’ contributions

JS and MJD conceived and designed the study. LK, CRM and JS conducted and interpreted the analyses. LK and JS drafted the manuscript. All authors reviewed and edited the manuscript and approve the final version.

## Supplementary Material

Additional file 1: Appendix 1 Confirmatory factor analysis path diagrams. **Appendix 2.** Known-groups validity. Click here for file
