# Supplementary material for: Psychometric validation of the Self-Care Inventory-Revised (SCI-R) in UK adults with type 2 diabetes using data from the AT.LANTUS Follow-on study
Source: Health Qual Life Outcomes. 2013 Feb 26;11:24. doi: 10.1186/1477-7525-11-24 (PMC3608221; doi:10.1186/1477-7525-11-24)
Supplement: Additional file 1: Appendix 1 — Confirmatory factor analysis path diagrams. Appendix 2. Known-groups validity. [file 1477-7525-11-24-S1.doc]

*Appendix 1: Confirmatory factor analysis path diagrams*

| *Path diagram 1: Unidimensional (14 items; excluding item 3)*    The path diagram shows that the majority of the items intended to make up the single factor SCI-R scale load at an acceptable level (except items 8, 13 and 15 which load <0.40). The RMSEA (.111), CFI (.663) and significant chi-square (412.493, p<0.001) suggest that the model is not a good fit to the data. | *Path diagram 2: Unidimensional (13 items; excluding items 3 & 13)*    The path diagram shows that the majority of the items intended to make up the single factor SCI-R scale load at an acceptable level (except items 2, 8 and 15 which load <0.40). The RMSEA (.118), CFI (.670) and significant chi-square (385.36, p<0.001) suggest that the model is not a good fit to the data |
| --- | --- |
| Path diagram 3: Unidimensional (12 items; excluding item 3, 13 & 15)    The path diagram shows that the majority of the items intended to make up the single factor SCI-R scale load at an acceptable level (except items 2, and 8 which load <0.40). The RMSEA (.125), CFI (.677) and significant chi-square (349.02, p<0.001) suggest that the model is not a good fit to the data | Path diagram 4: 2-factor model (15 items; including item 3)    The path diagram shows that the majority of the items intended to make up a 2-factor SCI-R scale load at an acceptable level (except items 8, 15, 3 and 13) which load <0.40). The RMSEA (.104), CFI (.662) and significant chi-square (427.50, p<0.001) suggest that the model is not a good fit to the data |
| Path diagram 5: 2-factor model (15 items; including item 3)    The path diagram shows that the majority of the items intended to make up a 2-factor SCI-R scale load at an acceptable level (except items 2, 15, 3, 8 and 13) which load <0.40). The RMSEA (.104), CFI (.660) and significant chi-square (429.53, p<0.001) suggest that the model is not a good fit to the data | *Path diagram 6: 2-factor model (13 items; excluding items 3 & 13)*    The path diagram shows that the majority of the items intended to make up a 2-factor SCI-R scale load at an acceptable level (except items 2, 15 and 8) which load <0.40). The RMSEA (.119), CFI (.669) and significant chi-square (385.36, p<0.001) suggest that the model is not a good fit to the data |

Appendix 2: Known-groups validity

| SCI-R | **Diabetes duration** | | **HbA1c** | | **Treatment**  **algorithm^+^** | | **Complications** | |
| --- | --- | --- | --- | --- | --- | --- | --- | --- |
|  | ≤16 years | >16 years | ≤7.5 (58mmol/  mol) | >7.5  (58mmol/  mol) | Algorithm  1 | Algorithm  2 | Present | Absent |
| n | 179 | 173 | 116 | 236 | 188 | 165 | 246 | 106 |
| 1. Check blood glucose with monitor | **4.02**  **(1.0)** | **4.51**  **(0.7)***** | **4.52**  **(0.8)** | **4.14**  **(1.0)**** | 4.22  (0.9) | 4.31  (0.9) | 4.33  (0.9) | 4.12  (1.1) |
| 2. Record blood glucose | **3.60**  **(1.4)** | **4.12**  **(1.1)**** | **4.09**  **(1.3)** | **3.75**  **(1.3)*** | 3.77  (1.4) | 3.95  (1.3) | 3.93  (1.3) | 3.69  (1.4) |
| 4. Correct dosage of pills/insulin | 4.79  (0.6) | 4.83  (0.5) | 4.84  (0.6) | 4.79  (0.6) | 4.77  (0.7) | 4.86  (0.4) | 4.80  (0.6) | 4.82  (0.5) |
| 5. Take pills/insulin at correct time | 4.60  (0.6) | 4.60  (0.7) | **4.73**  **(0.5)** | **4.54**  **(0.7)**** | 4.62  (0.7) | 4.58  (0.6) | 4.57  (0.7) | 4.67  (0.5) |
| 6. Correct food portions | 3.72  (0.8) | 3.83  (0.8) | 3.83  (0.8) | 3.75  (0.8) | 3.75  (0.8) | 3.80  (0.8) | 3.75  (0.8) | 3.83  (0.8) |
| 7. Meals/snacks eaten on time | **3.82**  **(0.9)** | **4.02**  **(0.7)*** | **4.13**  **(0.7)** | **3.82**  **(0.9)**** | 3.87  (0.9) | 3.98  (0.8) | 3.90  (0.9) | 3.97  (0.8) |
| 8. Keep food records | **1.49**  **(0.9)** | **1.70**  **(1.1)*** | 1.56  (1.0) | 1.61  (1.0) | 1.58  (1.0) | 1.61  (1.0) | 1.62  (1.0) | 1.52  (0.9) |
| 9. Read food labels | **2.95**  **(1.4)** | **3.30**  **(1.3)*** | 3.22  (1.5) | 3.08  (1.3) | 3.05  (1.4) | 3.21  (1.3) | 3.11  (1.4) | 3.16  (1.4) |
| 10. Recommended carbs | 3.44  (1.3) | 3.69  (1.2) | 3.75  (1.2) | 3.49  (1.3) | 3.54  (1.2) | 3.61  (1.3) | 3.58  (1.2) | 3.55  (1.3) |
| 11. Carry quick acting sugar | 3.72  (1.5) | 3.85  (1.5) | 3.96  (1.4) | 3.70  (1.4) | 3.73  (1.5) | 3.85  (1.4) | 3.80  (1.5) | 3.74  (1.5) |
| 12. Attend clinic appointments | 4.82  (0.5) | 4.90  (0.4) | **4.95**  **(0.2)** | **4.82**  **(0.6)*** | 4.87  (0.5) | 4.85  (0.4) | 4.85  (0.5) | 4.88  (0.4) |
| 13. Wear medic alert | 2.04  (1.7) | 2.27  (1.7) | 2.25  (1.8) | 2.11  (1.7) | 2.12  (1.7) | 2.19  (1.7) | 2.16  (1.7) | 2.13  (1.7) |
| 14. Exercise | 3.41  (1.1) | 3.29  (1.1) | 3.40  (1.0) | 3.32  (1.1) | 3.39  (1.1) | 3.31  (1.1) | 3.31  (1.1) | 3.45  (1.1) |
| 15. Adjust insulin dosage | 3.28  (1.5) | 3.47  (1.4) | 3.35  (1.5) | 3.39  (1.4) | 3.40  (1.4) | 3.35  (1.4) | 3.30  (1.4) | 3.58  (1.3) |
| SCI-R Total Score | **67.00**  **(13.0)** | **71.00**  **(12.2)***** | **72.00**  **(10.8)** | **68.00**  **(13.5)*** | 68.00  (13.5) | 70.00  (12.0) | 69.00  (13.3) | 69.00  (11.8) |

Data are mean (standard deviation)

+ Algorithm 1 (fixed insulin doses) of the original protocol; Algorithm 2 (self-titration) regardless of insulin type

*p<0.05, ** p<0.01, *** p<0.001 (Mann Whitney U-Tests)
